# Temporal trends and demographic-geographic disparities in kidney Cancer mortality in the United States, 1999–2023

**DOI:** 10.1016/j.pmedr.2025.103312

**Published:** 2025-11-13

**Authors:** Hui Gou, Zhilan Gan, Ping Chen, Xingxing Xie, Ling Fan, Jinwei Li, Wenbing Wu

**Affiliations:** a Department of Pharmacy, The Affiliated Hospital, Southwest Medical University, Luzhou 646000, China; b Department of Biochemistry and Molecular Biology, School of Basic Medical Sciences, Southwest Medical University, Luzhou 646000, China; c Department of Pharmacy, Suining Central Hospital, Suining 629000, China; d Department of Pharmacy, Ya'an People's Hospital, Ya'an 625000, China; e Drug Clinical Trial Institution, Ya'an People's Hospital, Ya'an 625000, China

**Keywords:** Kidney cancer, Mortality rate, Temporal trend, Health disparity, United States, Joinpoint regression

## Abstract

**Objective**: To analyze temporal trends and disparities in kidney cancer mortality in the United States (1999–2023) by demographics, geography, and urbanization, using data from the Centers for Disease Control and Prevention (CDC) WONDER database. **Methods**: Death data were extracted from the CDC Wonder database covering the United States during the period 1999–2023 and analyzed via Joinpoint regression and spatial analysis. **Results**: In 1999–2023, kidney cancer deaths increased by 33.38 % (from 10,862 to 14,488), while the age-adjusted mortality rate (AAMR) declined significantly (average annual percentage change (AAPC) = −0.76 %) in the United States. By age, 35–44 and 45–54-year-olds had the sharpest AAMR drops (annual percentage change (APC) = −1.81 and − 1.92, respectively); ≥75-year-olds rose, with the 85+ group's unadjusted mortality increasing annually (AAPC = 0.58 %). By sex, women's AAMR fell steadily (2005–2023 APC = -1.65), while men's stagnated after 2018. By race, Hispanics (AAPC = -0.76 %) and non-Hispanic Blacks (AAPC = -1.33 %) declined; non-Hispanic Whites stabilized later. Geographically, the Midwest showed “rise-fall-accelerated decline-stagnation” fluctuation, the West dropped steadily (AAPC = -0.91 %); metropolitan areas fell faster (2012–2020 APC = -1.97) than non-metros (AAPC = -0.39 %). **Conclusion**: In the United States, kidney cancer mortality declined overall but with significant disparities, requiring targeted interventions.

## Highlights

• Kidney cancer mortality declined in the United States from 1999 to 2023.
• Mortality dropped most in 35–54 age group but rose in ≥75.
• Mortality of females declined steadily, but males stagnated later.
• Hispanics and non-Hispanic Blacks had steady mortality decline.
• Urban areas had larger later mortality declines than rural areas.

## 1 Introduction

Kidney cancer ranks 12th globally in cancer mortality and accounts for approximately 2 % of cancer deaths in the United States, making it a key public health concern (Du et al., 2020; Lee et al., 2024; Doddi and Rashid, 2024; Mobaraki et al., 2024; Doddi and Rashid, 2024). Although advances in diagnostic imaging and effective systemic treatments have improved the prognosis for patients with kidney cancer, significant regional variations exist in the changing trends in kidney cancer mortality rates worldwide (Cai et al., 2020). Studies have shown that trends in kidney cancer mortality rates vary across 56 countries due to differences in medical conditions and diagnostic technologies (e.g., incidental findings from abdominal imaging such as computed tomography scans) (Sung et al., 2021). In the United States, cancer prevention and control data present unique trends. A study based on data from the American Cancer Society, the Centers for Disease Control and Prevention (CDC), the National Cancer Institute, and the North American Association of Central Cancer Registries from 2001 to 2019 showed that while the incidence of malignant tumors, including kidney cancer, continues to rise, mortality rates have shown a downward trend (Cronin et al., 2022). This trend was further confirmed by an analysis of data from the Surveillance, Epidemiology, and End Results database from 2000 to 2017. The study also noted that causes of death in kidney cancer patients include not only the cancer itself but also secondary malignancies (such as lung and bronchial cancer) and non-cancer conditions (such as heart disease and chronic obstructive pulmonary disease), and that significant regional and population-based disparities in kidney cancer-related deaths exist (Yang et al., 2023). For example, American Indians/Alaska Natives have significantly higher kidney cancer incidence and mortality rates than Whites, and these differences vary across sexes (Gonzalez et al., 2022). However, existing studies still have limitations: most studies lack sufficient stratified analyses, failing to fully explore differences across subgroups such as sex, race, and region. Furthermore, systematic analyses of key dimensions such as urbanization level and age groups have been insufficient, making the results less reflective of the actual disease burden across different populations (Ghazwani et al. Doddi and Rashid, 2024).

This study, based on the CDC WONDER database, systematically analyzed long-term trends in kidney cancer mortality in the United States from 1999 to 2023, focusing on differences across demographic characteristics (sex, race), geographic factors (state, census region, urban-rural classification), and age groups. Joinpoint regression models were used to quantify the timing and magnitude of these changes. The findings aim to provide data support for public health policymaking (such as targeted interventions for high-risk populations), uncover potential social determinants (such as access to healthcare resources and health inequalities) behind disparities in kidney cancer mortality, and complement the latest temporal trend evidence, providing guidance for subsequent etiological research and optimization of prevention and treatment strategies.

## 2 Materials and methods

### 2.1 Data sources and research objects

This study used data from the CDC WONDER Database, covering deaths from kidney cancer in the United States from 1999 to 2023. The international classification of disease 10 code for the disease is C64 (Malignant neoplasm of kidney, except renal pelvis). The study included all deaths from kidney cancer in the United States during this time period. Data were categorized by different dimensions, including demographic dimensions: sex (male/female), race (Hispanic, non-Hispanic black, non-Hispanic white, non-Hispanic other race), age group (decade interval groups: 25–34 years, 35–44 years, … 85 + years old); Geographic latitude: includes state (all 50 states plus the District of Columbia), census area (Northeast, Midwest, South, and West), and degree of urbanization (metropolitan/nonmetropolitan). This study was carried out based on the CDC WONDER public anonymous database, which has been granted an ethical review exemption by the Ethics Committee of Southwest Medical University (Reference Number: KY2022263) and complies with the security and privacy guidelines for the protection of human subjects. The Classification of urbanization was based on the 2023 Urban-Rural Classification Scheme for Counties from the U.S. Census Bureau, Specific information can refer to https://www.census.gov/programs-surveys/geography/guidance/geo-standards/urban-rural.html, The Census area classification is based on the four major census areas as defined by the U.S. Census Bureau, including the Northeast, Midwest, South and West.

### 2.2 Data analysis and statistics

The primary outcome measure was the age-adjusted mortality rate (AAMR, per 100,000 population), using direct standardization and using the year 2000 United States standard population as reference; unadjusted mortality rates were used for age-group analyses only. Temporal trends in mortality were assessed by Annual Percentage Change (APC) and Average Annual Percentage Change (AAPC). The time trend of mortality was analyzed, and the APC and 95 % confidence interval (CI) of each time period were calculated by separate subgroup analysis according to the degree of urbanization (metropolitan/non-metropolitan). The AAPC was used to reflect the overall change trend during the whole study period. A *P* value <0.05 was considered statistically significant. All data processing, statistical analysis, and visualization were performed using R (version 4.3.0; R Core Team, Vienna, Austria). The primary R packages used included usmap (mapping), ggpubr, and ggplot2 (visualization). The Joinpoint regression model (version 5.1.0.0; National Cancer Institute, Bethesda, Maryland, USA) was used for time trend analysis of mortality.

## 3 Results

### 3.1 Trends in renal cell carcinoma mortality in the United States, 1999–2023

From 1999 to 2023, the total number of kidney cancer deaths in the United States increased by 33.38 % (from 10,862 to 14,488), with the AAMR decreasing significantly from 6.15 (95 % CI: 6.03, 6.26) to 5.16 (95 % CI: 5.07, 5.24) and an average annual percentage change (AAPC) of −0.76 % (95 % CI: −1.07 to −0.45, *p* < 0.05). By sex, male deaths rose more substantially (43.86 % vs. 16.36 % in females), but females had a greater annual decline in AAMR (−1.28 % vs. -0.72 % in males, both significant). Geographically, the South saw the largest relative increase in deaths (53.20 %), while the Northeast had the most significant annual AAMR decline (−1.56 %, *p* < 0.05). Racial disparities were evident, with Hispanic and non-Hispanic Other groups experiencing the largest death increases (185.05 % and 176.32 %, respectively), and non-Hispanic Black individuals showing the steepest annual AAMR decrease (−1.33 %, p < 0.05). Metropolitan areas had a larger death increase (34.57 % vs. 28.66 % in nonmetropolitan areas) and a more significant annual AAMR decline (−1.20 % vs. -0.39 %, both significant). Age-wise, deaths decreased in younger groups (25–54 years) but increased in older groups, with the 85+ age group showing the largest death rise (81.47 %) and a significant annual increase in unadjusted mortality rate (0.58 %, p < 0.05), while the 45–54 age group had the steepest annual decline in unadjusted rate (−1.92 %, p < 0.05) (Table 1).

**Table 1:** Trends in the Number of deaths, age-adjusted mortality rate (AAMR, per 100,000 population), and average annual percentage change (AAPC) of kidney cancer in the American population, 1999–2023.

Characteristic	Deaths				AAMR		AAPC (95 % CI)
	1999	2023	Percent.change (%)		1999	2023	
Overall	10,862	14,488	33.38		6.2 (6.0, 6.3)	5.2 (5.1, 5.2)	-0.8 (−1.07, −0.45)
Sex							
Female	4139	4816	16.36		4.1 (3.9, 4.2)	3.2 (3.1, 3.2)	-1.3 (−1.53, −1.02)
Male	6723	9672	43.86		8.9 (8.7, 9.1)	7.6 (7.4, 7.7)	-0.7 (−1.12, −0.32)
Census Region							
Northeast	2137	2147	0.47		5.8 (5.6, 6.0)	4.2 (4.0, 4.3)	−1.6 (−2.17, −0.95)
Midwest	2780	3356	20.72		6.6 (6.4, 6.9)	5.7 (5.5, 5.9)	−0.7 (−1.48, 0.09)
South	3878	5941	53.20		6.2 (6.0, 6.4)	5.6 (5.4, 5.7)	−0.5 (−1.01, −0.06)
West	2067	3044	47.27		5.8 (5.6, 6.1)	4.8 (4.6, 4.9)	−0.9 (−1.02, −0.80)
Race							
Hispanic	535	1525	185.05		5.4 (4.9, 5.9)	5.0 (4.8, 5.3)	−0.8 (−1.01, −0.51)
non-Hispanic Black	1001	1359	35.76		6.4 (6.0, 6.8)	4.9 (4.6, 5.1)	−1.3 (−1.53, −1.13)
non-Hispanic White	9110	11,051	21.31		6.2 (6.1, 6.4)	5.5 (5.4, 5.6)	−0.6 (−1.01, −0.17)
non-Hispanic Other	190	525	176.32		3.6 (3.0, 4.1)	2.6 (2.4, 2.8)	−1.0 (−1.81, −0.21)
Urbanization							
Metropolitan	8681	11,682	34.57		6.0 (5.9, 6.1)	5.0 (4.9, 5.1)	−1.2 (−1.46, −0.94)
Nonmetropolitan	2181	2806	28.66		6.7 (6.4, 6.9)	6.4 (6.1, 6.6)	−0.4 (−0.56, −0.22)
Age							
25–34 years	54	49	−9.26		0.1 (0.0, 0.2)	0.1 (0.0, 0.2)	0.1 (−0.57, 0.83)
35–44 years	340	225	−33.82		0.8 (0.7, 0.9)	0.5 (0.4, 0.6)	−1.8 (−2.22, −1.41)
45–54 years	1139	840	−26.25		3.1 (2.9, 3.3)	2.1 (1.9, 2.3)	−1.9 (−2.15, −1.70)
55–64 years	2085	2565	23.02		8.8 (8.5, 9.1)	6.1 (5.8, 6.4)	−1.6 (−2.54, −0.64)
65–74 years	3012	4324	43.56		16.4 (16.0, 16.8)	12.5 (12.1, 12.9)	−1.4 (−1.92, −0.78)
75–84 years	2942	4144	40.86		24.1 (23.6, 24.6)	22.6 (22.1, 23.1)	−0.5 (−0.72, −0.19)
85+ years	1290	2341	81.47		31.1 (30.3, 31.9)	37.8 (36.9, 38.7)	0.6 (0.16, 1.00)

Notes: Statistical analysis: AAPCs and 95 % confidence intervals (CIs) were calculated using Joinpoint Regression Program. Urbanization level classification: Based on 2023 U.S. Census Bureau standards; AAMR data for 2023 urbanization levels are derived from 2020 records, with AAPCs calculated for 1999–2023. Age-stratified analysis: unadjusted mortality rates were used for age groups, with AAPCs calculated based on unadjusted rates. All AAPC values in the “AAPC (95 %CI)” column are statistically significant, except for the Midwest subgroup under the “Census Region” group and the 25–34 years subgroup under the “Age” group.

RCC, renal cell carcinoma; AAMR, age-adjusted mortality rate; CI, confidence interval; AAPC, average annual percentage change.

Overall, the data on kidney cancer deaths in the United States from 1999 to 2023 show a trend of total increase in the number of deaths but a general decrease in the mortality rate, and there are significant differences among different demographic subgroups.

### 3.2 Regional differences in kidney Cancer mortality in the United States, 1999–2023

**Fig. 1:**
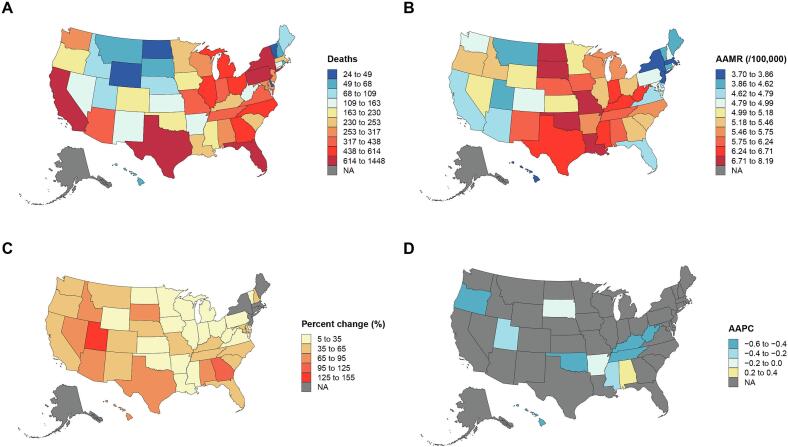
Geographic disparities in kidney cancer mortality burden, number of deaths, age-adjusted mortality rates, and temporal changes (percent change and average annual percent change) among the American population, 1999–2023. (A) Number of kidney cancer deaths per state in 2023. (B) Age-adjusted mortality rates per 100,000 population for kidney cancer per state in 2023. (C) Percent change in kidney cancer deaths per state from 1999 to 2023. (D) Average annual percent change in kidney cancer mortality per state from 1999 to 2023.

The burden of kidney cancer deaths in the United States from 1999 to 2023 showed significant geographic heterogeneity (Fig. 1). The number of kidney cancer deaths in 2023 varied significantly across states, ranging from 24 to 1448 cases, and showed a clear dependence on population size: high-burden states (≥438 deaths) were primarily concentrated in coastal and Midwestern regions, exemplified by California, Texas, and Florida, while low-burden states (≤68 deaths) were primarily located in mountainous areas and New England, such as Wyoming and Vermont (Fig. 1A).

The spatial distribution of AAMR varied significantly, ranging from 3.70 to 8.19 cases per 100,000 population. High mortality rates (≥6.24 per 100,000) were primarily located in the Midwest and Southern states, including North Dakota, Missouri, and Louisiana. Low mortality rates (≤4.62 per 100,000) were more common in the coastal West (e.g., California, Arizona) and the Northeast (e.g., Maine, Massachusetts) (Fig. 1B).

The percentage change in kidney cancer deaths across states from 1999 to 2023 generally showed an upward trend (5–155 %). States with rapid increases (≥65 %) were concentrated in the Southwest (e.g., Utah, Arizona, Nevada), the South (e.g., Georgia, Alabama), and the Rocky Mountain region (e.g., Idaho) (Fig. 1C).

Analysis of the average annual percentage change revealed a downward trend in mortality rates in all states, except Alabama (Fig. 1D).

In summary, the geographic heterogeneity of the kidney cancer mortality burden in the United States is significant: while death counts correlate with population size, AAMR disparities (independent of population) highlight regional differences in disease severity and healthcare access.

### 3.3 Trends and annual changes in kidney Cancer mortality by demographic characteristics in the United States, 1999–2023

**Fig. 2:**
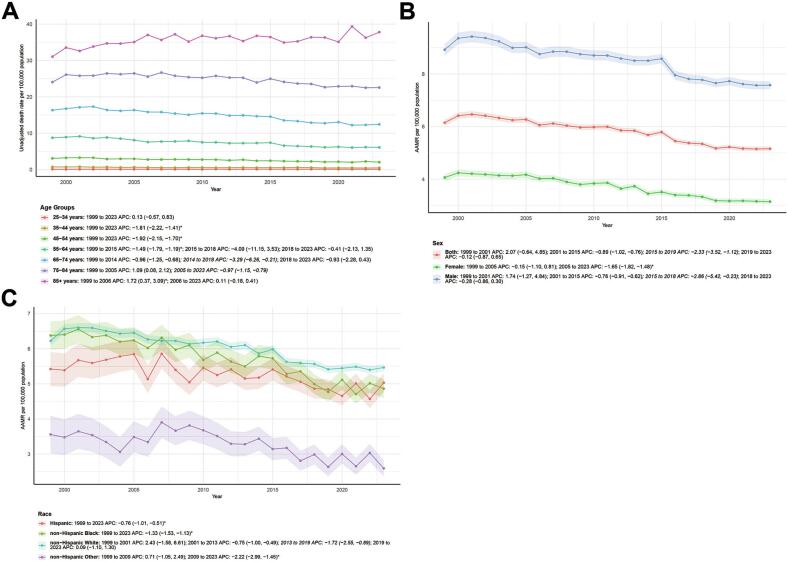
Temporal trends in kidney cancer mortality rates (unadjusted and age-adjusted). and annual percent changes by age, sex, and race among the American population, 1999–2023. (A) Unadjusted mortality rates for kidney cancer per 100,000 population across age groups. (B) Age-adjusted mortality rates for kidney cancer per 100,000 population by sex. (C) Age-adjusted mortality rates for kidney cancer per 100,000 population by race. Notes: AAMR, age-adjusted mortality rate; APC, annual percent change.

Temporal trends in unadjusted kidney cancer mortality rates across age groups showed a downward trend from 1999 to 2023, with the exception of those aged 75 years and older. The most significant decrease was observed in the 35–54 age group (APC = −1.81 for 35–44 years, APC = −1.92 for 45–54 years, both *P* < 0.05). While the overall trend was upward for those aged 75 years and older, the 75–84 age group showed a downward trend from 2005 to 2023 (APC = −0.97) (Fig. 2A).

Sex-stratified AAMR analysis showed downward trends in men, women, and the overall population, with differences in timing and magnitude. The overall population's AAMR increased from 1999 to 2001 (APC = 2.07, 95 % CI: −0.64, 4.85), decreased from 2001 to 2015 (APC = −0.89, 95 % CI: −1.02, −0.76), declined more sharply in 2015–2019 (APC = -2.33, 95 % CI: −3.52, −1.12), and nearly stagnated in 2019–2023 (APC = -0.12, 95 % CI: −0.87, 0.65). For females, AAMR slightly decreased from 1999 to 2005 (APC = -0.15, 95 % CI: −1.10, 0.81) and then significantly declined continuously from 2005 to 2023 (APC = -1.65, 95 % CI: −1.82, −1.48, P < 0.05). For males, AAMR increased from 1999 to 2001 (APC = 1.74, 95 % CI: −1.27, 4.84), decreased from 2001 to 2015 (APC = -0.76, 95 % CI: −0.91, −0.62), significantly declined in 2015–2018 (APC = -2.86, 95 % CI: −5.42, −0.23), and nearly stagnated in 2018–2023 (APC = -0.28, 95 % CI: −0.86, 0.30). Overall, both sexes showed AAMR declines after 2001, with women exhibiting a more sustained and significant downward trend (Fig. 2B).

There were significant differences in the trends of AAMR among different races from 1999 to 2023: Hispanics showed a continuous and significant decline (APC = -0.76, 95 % CI: −1.01, −0.51); non-Hispanic Blacks also showed a significant decline during the same period and the magnitude was greater (APC = −1.33, 95 % CI: −1.53, −1.13); non-Hispanic Whites showed a phased change, with a slight increase from 1999 to 2001 (APC = 2.43), a continuous decline after 2001 (a more significant decline from 2013 to 2019, APC = -1.72), and almost stagnant from 2019 to 2023 (APC = 0.09); non-Hispanic other races (primarily composed of American Indians/Alaska Natives and small populations of other ethnicities) showed a slight increase from 1999 to 2009 (APC = 0.71). After 2009, the AAMR showed a significant decline (APC = −2.22) (Fig. 2C).

Overall, the AAMR showed a downward trend for most ethnic groups, but the onset and magnitude varied: the decline continued steadily for Hispanics and non-Hispanic Blacks, declined significantly in non-Hispanic Others later, and plateaued in non-Hispanic Whites later. In summary, trends in kidney cancer mortality in the United States from 1999 to 2023 differed significantly by demographic characteristics (age, sex, and race). Although the overall trend was downward, the onset, magnitude, and persistence of the change varied across different groups.

### 3.4 Trends in age-adjusted kidney cancer mortality by census region and urbanization level in the United States, 1999–2023

**Fig. 3:**
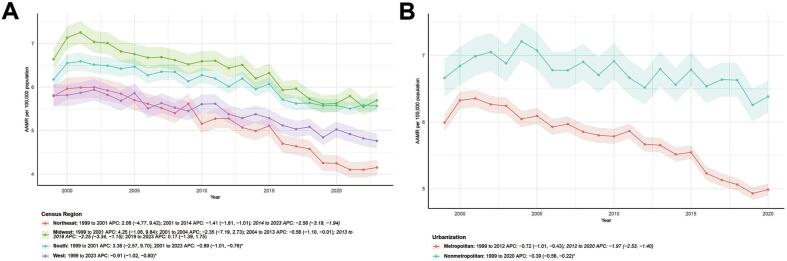
Temporal trends in kidney cancer age-adjusted mortality rates and annual percent changes by census regions and urbanization levels among the American population, 1999–2023. (A) Age-adjusted mortality rates for kidney cancer per 100,000 population by census regions. (B) Age-adjusted mortality rates for kidney cancer per 100,000 population by urbanization levels. Notes: Census areas were based on the official U.S. Census Bureau classification criteria, the classification of urbanization levels was based on the 2023 urban-Rural classification scheme of the United States Census Bureau. AAMR, age-adjusted mortality rate; APC, annual percent change.

Censuses stratified by region revealed regional differences in AAMR trends for kidney cancer, based on the four official U.S. Census Bureau regions: in the Northeast, the trend was upward from 1999 to 2001 (APC = 2.08, 95 % CI: −4.77, −9.42), and continued to decline after 2001, with a decrease of −2.56 from 2014 to 2023. In the Midwest, the trend was “rise-fall-accelerated decline-stagnation”, with an accelerated increase from 1999 to 2001 (APC = 4.25, 95 % CI: −1.06, 9.84), a continuous decline from 2001 to 2019, and stagnation after 2019 (APC = 0.17, 95 % CI: −1.39, 1.75). In the South, the trend was upward from 1999 to 2001 (APC = 3.38, 95 % CI: −2.57, 9.70), and then decreased from 2001 to 2023 (APC = -0.89, 95 % CI: −1.01, −0.76). In the West, the AAMR maintained a stable and significant downward trend from 1999 to 2023 (APC = −0.91, *P* < 0.05) (Fig. 3A).

In summary, U.S. kidney cancer age-adjusted mortality trends varied markedly by census region: the Northeast declined sustainably post-2001; the Midwest showed a unique four-stage “rise-fall-accelerated decline-stagnation” pattern; the South decreased gradually after 2001; the West maintained a stable, significant long-term decline.

### 3.5 Trends in age-adjusted kidney cancer mortality by urbanization level in the United States, 1999–2023

Urbanization-stratified analysis showed that metropolitan areas' AAMR slightly declined from 1999 to 2012 (APC = −0.72, 95 % CI: −1.01, −0.43), with a significant acceleration in the decline rate from 2012 to 2020 (APC = -1.97, P < 0.05). In nonmetropolitan areas, AAMR declined significantly and uniformly from 1999 to 2020 (APC = -0.39, P < 0.05), but at a slower pace than in metropolitan areas (Fig. 3B). In summary, urban-rural differences in AAMR lead to differentiated mortality declines (accelerated late-period decline in urban areas), highlighting the potential regulatory role of urban-rural factors in kidney cancer mortality. This gap may stem from concentrated medical resources in urban areas (e.g., more radiologists, better access to targeted therapies), whereas rural areas face challenges like limited diagnostic capacity and delayed treatment.

## 4 Discussion

This study showed an overall decrease in the AAMR for kidney cancer in the U.S. from 1999 to 2023 (AAPC = −0.76 %), consistent with a CDC Wonder database study reporting a − 0.6 % AAPC for 1999–2020 (DODDI and RASHID Doddi and Rashid, 2024). This decline may relate to earlier diagnosis via widespread abdominal imaging (e.g., computed tomography scans), which detects asymptomatic kidney tumors incidentally (Shehata et al., 2021) and clinical use of targeted therapies (e.g., vascular endothelial growth factor inhibitors) and immunotherapies (Ding et al., 2021; Pal et al., 2019). However, mortality decline (−0.76 % annual average) was slower than incidence increase (+1.5 % annual average), possibly due to “treatment disconnect”, a term first proposed by Turner (Turner et al., 2017), referring to the gap between advances in treatment and incomplete translation to mortality reduction in specific populations, suggesting limitations from late-stage case growth or insufficient treatment efficacy.

Age-stratified results revealed the most significant mortality decline in 35–54-year-olds (APC = −1.81 to −1.92), while those ≥75 years showed an upward trend. Younger groups may access more preventive care (e.g., physicals) and have less exposure to risks (e.g., smoking, obesity). A 2001–2008 retrospective analysis of 316 renal cell carcinoma patients showed under-40s had higher non-clear cell tumor rates, metastasis rates, and lower 5-year survival (Lee et al., 2011). Elderly individuals had poorer prognoses due to complex comorbidities and low treatment tolerance (Calaway et al., 2018; Gao et al., 2018). Notably, the mortality rate in the 75–84 age group decreased after 2005 (APC = −0.97), consistent with the findings of Doddi et al. (DODDI and RASHID, 2024), hypothesized to link to stabilized incidence and widespread targeted therapies (Chow and Devesa, 2008).

For sex differences, women's renal cell carcinoma AAMR declined consistently (2005–2023 APC = −1.65), while men's stagnated post-2018. This may stem from sex-specific risks: men have higher smoking rates and occupational exposures (e.g., chemicals), leading to worse incidence, tumor aggressiveness, prognosis, and treatment response (Muscat, 2000). Women may have different risks/prognoses due to hormonal (e.g., estrogen) and genetic factors (e.g., X-chromosome inactivation escape) (Mariangela et al., 2020; Peired et al., 2021).

Among racial disparities, Hispanics and non-Hispanic Blacks had continued mortality decline, while non-Hispanic Whites stabilized later. This may reflect socioeconomic status (SES): Hispanics and non-Hispanic Blacks have more low-to-middle-income groups (Mafolasire et al., 2016), and improved healthcare access (e.g., Affordable Care Act) narrowed treatment gaps (Kish et al., 2014). Non-Hispanic Whites' stagnation may link to saturated abdominal imaging detection or poor risk control (e.g., obesity) (Siegel et al., 2018). Notably, non-Hispanic Other population mortality dropped significantly post-2009 (APC = −2.22), possibly due to small population base and pronounced policy effects.

Regional results showed the Midwest had four-stage fluctuations (“rise-fall-accelerated decline-stagnation”), linked to industrialization-related environmental exposure (e.g., air pollution) and medical resource changes (An and Liu, 2021; Ghazwani et al., 2024; Turner et al., 2017). The South had continuous decline (APC = −0.89) via tobacco control (e.g., higher taxes) and widespread use of abdominal imaging (facilitating incidental detection of early-stage kidney cancer). These specific drivers clarify that future studies should test whether targeted interventions—such as stricter industrial emission standards in the Ohio Valley or expanded tele-oncology in rural Midwest areas—can reverse post-2019 stagnation. The West had stable long-term decline (APC = −0.91) due to high-income concentration and strong health awareness (Siegel et al., 2024, Siegel et al., 2016).

For urban-rural differences, urban areas had larger mortality decline in 2012–2020 (APC = −1.97) via standardized diagnosis/treatment and concentrated primary care (e.g., more radiologists, lower misdiagnosis, better targeted therapy access) (Semprini et al., 2024; Sokale et al., 2023). Non-urban areas declined slower (APC = −0.39), requiring strengthened rural detection via abdominal imaging (e.g., mobile medical vehicles) and remote pathology support (Charlton et al., 2015; Cosby et al., 2019).

Limitations of this study include: ① The CDC Wonder database lacks individual-level SES data, which prevents us from analyzing how factors like poverty or educational attainment mediate mortality disparities (e.g., whether rural-urban gaps are driven by SES rather than geographic access alone) (Salichs et al., 2023); ② Inability to distinguish kidney cancer subtype impacts (e.g., clear cell vs. non-clear cell) (Costa et al., 2007); ③ Incomplete 2023 data affecting latest trend accuracy. Future studies should integrate electronic health records and molecular pathology to analyze SES-genetic susceptibility (e.g., Von Hippel-Lindau mutations)-mortality links, evaluate new diagnostic tools for early detection (e.g., urine metabolomics) in high-risk groups, and explore AI-assisted diagnosis in rural areas (Sankin et al., 2014).

In conclusion, U.S. kidney cancer mortality decline results from combined diagnostic imaging technologies, treatment, and public health policies. Subgroup differences highlight health inequality complexity; future efforts need precision medicine and policy innovation to achieve universal cancer prevention/control access.

Notes: AAMR, age-adjusted mortality rate; AAPC, average annual percentage change.

## CRediT authorship contribution statement

**Hui Gou:** Writing – original draft, Visualization, Funding acquisition. **Zhilan Gan:** Writing – original draft, Methodology. **Ping Chen:** Writing – original draft, Software, Formal analysis. **Xingxing Xie:** Writing – original draft, Validation, Software. **Ling Fan:** Writing – original draft, Validation, Software. **Jinwei Li:** Writing – review & editing, Resources, Project administration, Conceptualization. **Wenbing Wu:** Writing – review & editing, Project administration, Funding acquisition.

## Funding sources

This study was supported by The Science and Technology Strategic Cooperation Programs of Luzhou Municipal People's Government and Southwest Medical University (2024LZXNYDJ096), Southwest Medical University (00170015), (2024ZKY008).

## Declaration of competing interest

The authors declare that they have no known competing financial interests or personal relationships that could have appeared to influence the work reported in this paper.

## Data Availability

Data will be made available on request.

## Appendix A Supplementary data

Supplementary material Image 1
