# Supplementary material for: Temporal trends and demographic-geographic disparities in kidney Cancer mortality in the United States, 1999–2023
Source: Prev Med Rep. 2025 Nov 13;60:103312. doi: 10.1016/j.pmedr.2025.103312 (PMC12664346; doi:10.1016/j.pmedr.2025.103312)
Supplement: Supplementary file 1 — Supplementary material [file mmc1.pdf]

西南医科大学附属医院临床试验伦理委员会批文

受理号：KY2022263

项目名称	低表达Nebulette与肾透明细胞癌不良预后及免疫细胞浸润缺陷有关		
项目来源	四川省医学会青年创新项目		
研究单位	西南医科大学附属医院		
承担科室	泌尿外科	主要研究者	程波
审查类别	初始审查	审查方式	□全会 ☑快速 □紧急
审查日期	2022年09月26日	审查地点	NA

审查文件：

☑ 研究方案（版本号：1.0； 版本日期：2022.08.15）

☑ 免除签署知情同意书（版本号：NA）

审查意见：

根据ICH-GCP、中国GCP及相关法律、法规的规定，经本伦理委员会审查，同意按所批准的临床研究方案、知情同意书开展本项研究。

研究过程中注意事项（请仔细阅读）：

1.请遵循GCP原则，自觉接受国家有关法律和法规约束，遵循伦理委员会批准的方案开展临床研究，保护受试者的健康与权利；2.研究开始前，请申请人完成临床试验注册；3.所有资料未经伦理委员会批准，不得做任何修改；4. 从批准之日起，应每年向本伦理委员会提交年度/定期跟踪审查报告，请在持续审查日期到期前一个月提出持续审查的申请；5. 试验过程中发生以下情况应及时报告：①发生任何严重不良事件请立即（24小时内）报告；②违反研究方案；③暂停/终止研究；6. 研究完成后提交总结报告；7.本批件自批准之日起一年内有效，逾期则自动废止。

审查结论：同意。

有效期	1年	跟踪审查频率	12个月

地址：泸州市太平街25号　邮编：646000　联系人：张增瑞　电话：0830-3165273

伦理委员会主任委员（签章）：陈正君　西南医科大学附属医院临床试验伦理委员会（盖章）

2022年09月26日
